# Supplementary material for: Modeling glioblastoma heterogeneity as a dynamic network of cell states
Source: Mol Syst Biol. 2021 Sep 16;17(9):e10105. doi: 10.15252/msb.202010105 (PMC8444284; doi:10.15252/msb.202010105)
Supplement: Supplementary file 6 — Source Data for Figure 5 [file MSB-17-e10105-s004.zip › Figure5A_sourcedata/GSEA_3071/hallmarks_stateIII.GseaPreranked.1623742540963/HALLMARK_ESTROGEN_RESPONSE_EARLY.html]

Details for gene set HALLMARK\_ESTROGEN\_RESPONSE\_EARLY[GSEA]

|  || Dataset | state33071 |
| Phenotype | NoPhenotypeAvailable |
| Upregulated in class | na\_neg |
| GeneSet | HALLMARK\_ESTROGEN\_RESPONSE\_EARLY |
| Enrichment Score (ES) | -0.3783971 |
| Normalized Enrichment Score (NES) | -2.0759187 |
| Nominal p-value | 0.0 |
| FDR q-value | 0.003988191 |
| FWER p-Value | 0.035 |
Table: GSEA Results Summary

  

Fig 1: Enrichment plot: HALLMARK\_ESTROGEN\_RESPONSE\_EARLY      
 Profile of the Running ES Score & Positions of GeneSet Members on the Rank Ordered List

  

| PROBE | GENE SYMBOL | GENE\_TITLE | RANK IN GENE LIST | RANK METRIC SCORE | RUNNING ES | CORE ENRICHMENT || 1 | MYC |  |  | 599 | -0.260 | -0.1447 | No |
| 2 | FKBP5 |  |  | 657 | -0.263 | -0.1492 | No |
| 3 | UNC119 |  |  | 667 | -0.263 | -0.1412 | No |
| 4 | ALDH3B1 |  |  | 822 | -0.270 | -0.1705 | No |
| 5 | KLF4 |  |  | 973 | -0.278 | -0.1984 | No |
| 6 | SLC19A2 |  |  | 1096 | -0.284 | -0.2188 | No |
| 7 | TMEM164 |  |  | 1222 | -0.291 | -0.2398 | No |
| 8 | TIAM1 |  |  | 1276 | -0.294 | -0.2420 | No |
| 9 | ADCY9 |  |  | 1286 | -0.294 | -0.2328 | No |
| 10 | JAK2 |  |  | 1401 | -0.300 | -0.2506 | No |
| 11 | INPP5F |  |  | 1558 | -0.310 | -0.2788 | No |
| 12 | TOB1 |  |  | 1573 | -0.310 | -0.2703 | No |
| 13 | MICB |  |  | 1576 | -0.311 | -0.2586 | No |
| 14 | CALB2 |  |  | 1639 | -0.315 | -0.2624 | No |
| 15 | SEC14L2 |  |  | 1726 | -0.320 | -0.2721 | No |
| 16 | ABLIM1 |  |  | 1940 | -0.332 | -0.3142 | No |
| 17 | SLC2A1 |  |  | 1961 | -0.333 | -0.3063 | No |
| 18 | SLC16A1 |  |  | 2012 | -0.336 | -0.3061 | No |
| 19 | SLC39A6 |  |  | 2054 | -0.338 | -0.3035 | No |
| 20 | XBP1 |  |  | 2097 | -0.341 | -0.3010 | No |
| 21 | RRP12 |  |  | 2257 | -0.353 | -0.3283 | No |
| 22 | WFS1 |  |  | 2307 | -0.358 | -0.3270 | No |
| 23 | GJA1 |  |  | 2362 | -0.362 | -0.3268 | No |
| 24 | WWC1 |  |  | 2385 | -0.364 | -0.3183 | No |
| 25 | CD44 |  |  | 2471 | -0.371 | -0.3258 | No |
| 26 | ABAT |  |  | 2521 | -0.375 | -0.3238 | No |
| 27 | STC2 |  |  | 2570 | -0.379 | -0.3214 | No |
| 28 | DHCR7 |  |  | 2654 | -0.386 | -0.3277 | No |
| 29 | FHL2 |  |  | 2851 | -0.403 | -0.3626 | Yes |
| 30 | ELOVL2 |  |  | 2879 | -0.406 | -0.3537 | Yes |
| 31 | PEX11A |  |  | 2909 | -0.409 | -0.3452 | Yes |
| 32 | SVIL |  |  | 2912 | -0.409 | -0.3298 | Yes |
| 33 | TGM2 |  |  | 2933 | -0.413 | -0.3188 | Yes |
| 34 | SIAH2 |  |  | 2946 | -0.414 | -0.3057 | Yes |
| 35 | RHOBTB3 |  |  | 2955 | -0.415 | -0.2916 | Yes |
| 36 | SCARB1 |  |  | 2975 | -0.417 | -0.2802 | Yes |
| 37 | BHLHE40 |  |  | 2981 | -0.417 | -0.2652 | Yes |
| 38 | SLC1A4 |  |  | 3051 | -0.426 | -0.2664 | Yes |
| 39 | CHPT1 |  |  | 3060 | -0.426 | -0.2518 | Yes |
| 40 | TIPARP |  |  | 3072 | -0.428 | -0.2379 | Yes |
| 41 | BCL2 |  |  | 3081 | -0.429 | -0.2233 | Yes |
| 42 | PODXL |  |  | 3133 | -0.435 | -0.2194 | Yes |
| 43 | NAV2 |  |  | 3180 | -0.441 | -0.2141 | Yes |
| 44 | GREB1 |  |  | 3240 | -0.449 | -0.2118 | Yes |
| 45 | FAM102A |  |  | 3271 | -0.454 | -0.2018 | Yes |
| 46 | ENDOD1 |  |  | 3330 | -0.467 | -0.1985 | Yes |
| 47 | CELSR2 |  |  | 3378 | -0.475 | -0.1921 | Yes |
| 48 | SLC26A2 |  |  | 3382 | -0.476 | -0.1743 | Yes |
| 49 | OLFM1 |  |  | 3398 | -0.480 | -0.1594 | Yes |
| 50 | TFF3 |  |  | 3413 | -0.483 | -0.1442 | Yes |
| 51 | ADCY1 |  |  | 3492 | -0.505 | -0.1446 | Yes |
| 52 | OLFML3 |  |  | 3503 | -0.509 | -0.1273 | Yes |
| 53 | UGCG |  |  | 3532 | -0.521 | -0.1142 | Yes |
| 54 | TUBB2B |  |  | 3594 | -0.544 | -0.1087 | Yes |
| 55 | RETREG1 |  |  | 3618 | -0.560 | -0.0928 | Yes |
| 56 | FOS |  |  | 3621 | -0.561 | -0.0714 | Yes |
| 57 | TPBG |  |  | 3638 | -0.570 | -0.0532 | Yes |
| 58 | PRSS23 |  |  | 3658 | -0.584 | -0.0353 | Yes |
| 59 | CA12 |  |  | 3694 | -0.611 | -0.0205 | Yes |
| 60 | MPPED2 |  |  | 3736 | -0.655 | -0.0055 | Yes |
| 61 | NBL1 |  |  | 3743 | -0.664 | 0.0189 | Yes |
| 62 | BCL11B |  |  | 3799 | -0.740 | 0.0336 | Yes |
Table: GSEA details [plain text format]

  

Fig 2: HALLMARK\_ESTROGEN\_RESPONSE\_EARLY: Random ES distribution      
 Gene set null distribution of ES for **HALLMARK\_ESTROGEN\_RESPONSE\_EARLY**
